# Recruiting Adolescents With Chronic Fatigue Syndrome/Myalgic Encephalomyelitis to Internet-Delivered Therapy: Internal Pilot Within a Randomized Controlled Trial

**DOI:** 10.2196/17768

**Published:** 2020-08-12

**Authors:** Emma Anderson, Roxanne Parslow, William Hollingworth, Nicola Mills, Lucy Beasant, Daisy Gaunt, Chris Metcalfe, David Kessler, John Macleod, Susan Pywell, Kieren Pitts, Simon Price, Paul Stallard, Hans Knoop, Elise Van de Putte, Sanne Nijhof, Gijs Bleijenberg, Esther Crawley

**Affiliations:** 1 Centre for Academic Child Health Bristol Medical School: Population Health Sciences University of Bristol Bristol United Kingdom; 2 Bristol Medical School: Population Health Sciences University of Bristol Bristol United Kingdom; 3 Bristol Trials Centre University of Bristol Bristol United Kingdom; 4 Advanced Computing Research Centre University of Bristol Bristol United Kingdom; 5 Research IT University of Bristol Bristol United Kingdom; 6 Department of Computer Science University of Bristol Bristol United Kingdom; 7 Department for Health University of Bath Bath United Kingdom; 8 Department of Medical Psychology Amsterdam University Medical Centres, Amsterdam Public Health Research Institute University of Amsterdam Amsterdam Netherlands; 9 Department of Paediatrics, Wilhelmina Children's Hospital University Medical Centre Utrecht Utrecht Netherlands; 10 Radboud University Medical Center Nijmegen Netherlands

**Keywords:** pediatrics, chronic fatigue syndrome, myalgic encephalomyelitis, cognitive behavioral therapy, eHealth, online systems, e-therapy, e-counseling, pilot projects, qualitative research

## Abstract

**Background:**

Chronic fatigue syndrome/myalgic encephalomyelitis (CFS/ME) in adolescents is common and disabling. Teenagers in the United Kingdom are more likely to recover if they access specialist care, but most do not have access to a local specialist CFS/ME service. Delivering treatment remotely via the internet could improve access to treatment.

**Objective:**

This study aims to assess (1) the feasibility of recruitment and retention into a trial of internet-delivered specialist treatment for adolescents with CFS/ME and (2) the acceptability of trial processes and 2 web-based treatments (to inform continuation to full trial).

**Methods:**

This study is an internal pilot for the initial 12 months of a full randomized controlled trial (RCT), with integrated qualitative methods (analysis of recruitment consultations and participant and clinician interviews). Recruitment and treatment were delivered remotely from a specialist pediatric CFS/ME treatment service within a hospital in South West United Kingdom. Adolescents (aged 11-17 years) from across the United Kingdom with a diagnosis of CFS/ME and no access to local specialist treatment were referred by their general practitioner to the treatment center. Eligibility assessment and recruitment were conducted via remote methods (telephone and on the web), and participants were randomized (via a computer-automated system) to 1 of 2 web-based treatments. The trial intervention was Fatigue in Teenagers on the InterNET in the National Health Service, a web-based modular CFS/ME-specific cognitive behavioral therapy program (designed to be used by young people and their parents or caregivers) supported by individualized clinical psychologist electronic consultations (regular, scheduled therapeutic message exchanges between participants and therapist within the platform). The comparator was Skype-delivered activity management with a CFS/ME clinician (mainly a physiotherapist or occupational therapist). Both treatments were intended to last for up to 6 months. The primary outcomes were (1) the number of participants recruited (per out-of-area referrals received between November 1, 2016, to October 31, 2017) and the proportion providing 6-month outcome data (web-based self-report questionnaire assessing functioning) and (2) the qualitative outcomes indicating the acceptability of trial processes and treatments.

**Results:**

A total of 89 out of 150 (59.3% of potentially eligible referrals) young people and their parents or caregivers were recruited, with 75 out of 89 (84.2%) providing 6-month outcome data. Overall, web-based treatment was acceptable; however, participants and clinicians described both the advantages and disadvantages of remote methods. No serious adverse events were reported.

**Conclusions:**

Recruiting young people (and their parents or caregivers) into an RCT of web-based treatment via remote methods is feasible and acceptable. Delivering specialist treatment at home via the internet is feasible and acceptable, although some families prefer to travel across the United Kingdom for face-to-face treatment.

**Trial Registration:**

ISRCTN 18020851; http://www.isrctn.com/ISRCTN18020851

**International Registered Report Identifier (IRRID):**

RR2-10.1186/s13063-018-2500-3

## Introduction

Between 1% and 2.4% of children and teenagers are estimated to have chronic fatigue syndrome or myalgic encephalomyelitis (CFS/ME) [1,2]. Affected children and teenagers can have severe disability [3,4], and most of them have no access to specialist treatment in their locality. This situation forces children and teenagers to either remain untreated or travel long distances to access specialist care, which may exacerbate symptoms.

One solution is to provide specialist treatment remotely to enable all families in the United Kingdom to access treatment and remove the need for travel. A Dutch trial of a web-based CFS/ME-specific cognitive behavioral therapy (CBT) called Fatigue in Teenagers on the InterNET (FITNET) showed promising results [5]. Two-thirds of young people offered the intervention recovered at 6 months compared with just 8% in the control (usual care) arm.

However, the United Kingdom has a different system of health care provision, and further testing is required to investigate whether FITNET is effective and cost-effective in the National Health Service (NHS). In contrast to the Dutch study, the Fatigue in Teenagers on the InterNET in the National Health Service (FITNET-NHS) trial uses an active specialist treatment comparison group (activity management [AM] via Skype), rather than treatment as usual or waiting list control, as specifically recommended for this trial by the funders.

This pilot study aimed to assess the feasibility of the remote recruitment of adolescents (and their parents/caregivers) into a randomized controlled trial (RCT) of a UK-adapted version of the Dutch CBT program—FITNET-NHS—compared with a version of usual care—AM (delivered via Skype), and to assess the acceptability of the 2 web-based interventions.

## Methods

### Ethics

The pilot and full RCT protocol and all associated documents were reviewed and approved by the South West-Frenchay Research Ethics Committee (reference 16/SW/0268).

### Design and Recruitment

This study presents the findings of the initial 12-month internal pilot phase of the FITNET-NHS RCT, which used entirely remote methods to recruit, randomize, and treat adolescents who were referred to a specialist pediatric CFS/ME treatment service within a hospital in South West United Kingdom between November 1, 2016, to October 31, 2017.

The eligibility criteria included the following: (1) young people aged between 11 and 17 years, with (2) a diagnosis of CFS/ME, and (3) no access to local specialist pediatric CFS/ME treatment (defined as more than 1 hour’s journey to their closest specialist treatment center or >6 months’ waiting list). Exclusions were as follows: (1) patients whose fatigue was due to another cause or was not disabling, (2) patients who would be unable to complete video calls or web-based modules (eg, due to developmental problems, lack of literacy, or lack of internet access), or (3) patients who were pregnant at the time of assessment.

The young person’s parent/caregiver was asked to provide consent for the study. Additionally, participants aged between 11 and 15 years provided assent, whereas those aged between 16 and 17 years provided their consent to participate in the study.

The South West United Kingdom specialist pediatric CFS/ME treatment center has always accepted out-of-area (as well as local) referrals of children and young people from general practitioners (GPs) across the United Kingdom for both diagnosis and treatment of CFS/ME. This meant that many families had to travel long distances to the center for treatment. The FITNET-NHS trial was launched with a message to GPs in the United Kingdom that out-of-area referrals could now receive treatment without travel.

A detailed description of the study methods is presented elsewhere [6]. In brief, all referrals from GPs were screened by administrative staff, and those potentially eligible for FITNET-NHS were contacted by research nurses. The research nurses conducted an initial brief telephone discussion with the families of potentially eligible adolescents and provided information about the trial (including emailing patient information leaflets). For interested families, a second call by a research nurse was arranged to conduct full eligibility assessment, recruitment discussion, and take consent (via a web-based form). After consenting, participants were randomly allocated (individually via an automated web randomization service, set up and managed by Bristol Randomised Trials Collaboration to maintain allocation concealment) on a 1:1 ratio (using minimization to facilitate balance by age and gender) to 1 of the 2 interventions. Due to the nature of the interventions, it was not practical to blind the participant, family, or clinical service to treatment allocation. Participants and parents/caregivers completed a web-based (emailed) self-report set of baseline questionnaires, and all were followed up with (emailed) web-based questionnaires at 3, 6, and 12 months after recruitment.. A subsample was invited to be interviewed (qualitative recruitment details are provided under *Integrated Qualitative Methods* below). The 6-month web-based self-report Short Form Health Survey Physical Function Subscale (SF-36-PFS) 10-item questionnaire [7] is the primary outcome for the full trial. The SF-36-PFS measures disability, an important outcome for children with CFS/ME [8], and is sufficiently sensitive in this patient group.

### Outcome Measures

The primary outcomes for this pilot study were the number of eligible adolescents (and their parents/caregivers) recruited compared with the number referred before October 31, 2017, and the proportion of those providing 6-month web-based self-report outcome data (completion of SF-36-PFS). Key qualitative outcomes were acceptability of trial processes and treatments, identified through thematic analysis of interview data.

### Intervention Descriptions

#### FITNET-NHS

This is a web-based modular CFS/ME-specific CBT program designed to be used by young people and their parents or caregivers. It is supported by individualized clinical psychologist e-consultations (with messages sent separately to the young person and their parent or caregiver) delivered within the program itself. Each participant and their parent or caregiver are set up on the platform by the therapist once allocated to this treatment. The participant and parent or caregiver then each set up an independent password-protected log-in. There are up to 19 chapters for young people to work through, which are unlocked by the psychologist on completion of the previous chapter. Some chapters are optional and are only unlocked if the psychologist thinks they are relevant for the young person (eg, a chapter looking more closely at mood problems). The earlier chapters include explanations of the links between thoughts, feelings, and behavior that form patterns contributing to the maintenance of CFS/ME symptoms. The chapters include questions for young people to complete, designed to identify unhelpful patterns and help with problem-solving. Young people are encouraged to monitor their activity, establish a manageable baseline, and build on this gradually. There are diaries included in the program for young people to record their sleep, activity levels, and helpful thoughts, which they can then discuss with therapists. Parents or caregivers can read the content of the chapters but not the answers to the questions. Therapists can view participants’ question responses and diaries and they use tailored e-consultations (via message function within the platform) to help the young person through the course. Therapists request that the patient responds before an arranged appointment date within which the therapist will deliver their next detailed and tailored message. Treatment was designed to last approximately 6 months.

#### AM Via Skype

This is the comparison treatment, delivered by a CFS/ME clinician (usually a physiotherapist/occupational therapist). AM is a standard behavioral treatment offered within the specialist service and recommended by the National Institute for Health and Care Excellence [9]. It involves assessment of the young person’s activity level and begins with establishing a manageable baseline of activity to be maintained daily (usually reduction of activity) from which to build gradually and safely at a pace that the patient can manage. The AM intervention offered within the trial is protocolized and explicitly prohibits detailed engagement with cognitions, keeping it as a behavioral treatment. A total of 3 AM sessions were offered as a version of usual care for comparison with the FITNET-NHS intervention. However, in response to feedback, this was increased to 6 sessions from November 2017 onward. Although standard care is usually delivered face-to-face, Skype delivery of at least some of the AM sessions is becoming more routine within the service. For this trial, every aspect was delivered remotely. Treatment was designed to last approximately 6 months.

### Integrated Qualitative Methodology

We undertook one-off in-depth interviews with participants and their parents to understand their experiences and views of trial processes. The interviews assessed the provision and acceptability of patient information, treatment preferences, and acceptability of both the content and the delivery of treatments. Participants were purposively selected for maximum variation (intervention, age, and gender) [10]. Families were given a choice of being interviewed over Skype or telephone, together or alone.

Trial staff (recruiters and therapists) for both treatment arms were also interviewed to ascertain their views on the provision of trial information, how the trial treatment compares with standard care, the feasibility of delivering the intervention to children, their view of treatment effectiveness, and potential changes to the interventions offered. Interviews followed a checklist of topics to ensure that key areas were explored but were sufficiently flexible to allow new issues of importance to the participants to emerge. All interviews were audio recorded with consent using encryption software, transcribed verbatim, and anonymized.

We additionally audio recorded (with consent) the recruitment consultations (second call from research nurses) to identify areas for improvement to optimize recruitment and informed consent in the trial via research nurse training (results to be presented in a separate publication) [11,12].

### Qualitative Data Analysis

Qualitative data analysis was ongoing and iterative, commencing soon after data collection to inform further data collection [13]. Audio recordings were transcribed verbatim, imported into NVivo (QSR International) software, systematically assigned codes, and analyzed thematically using techniques of constant comparison [14]. For the in-depth interviews with families and therapists, the data were examined for patterns and themes, comparing accounts between different participants, and refining the coding framework as interviews progressed. To check coding reliability, one-fourth of the transcripts were double coded by other members of the team, and the findings were compared. Sources of difficulties identified through the qualitative data were discussed with the trial management group to improve aspects of the design, conduct, organization, or training of recruiters.

## Results

### Recruitment and Retention

**Figure 1:**
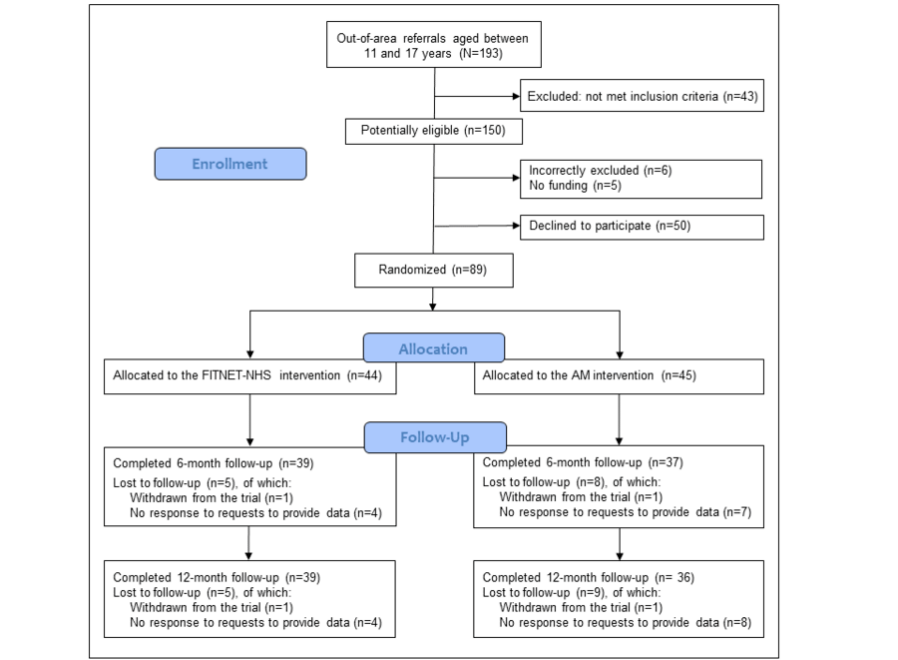
Consolidated Standards of Reporting Trials flow diagram. AM: activity management; FITNET-NHS: Fatigue in Teenagers on the InterNET in the National Health Service.

A total of 193 out-of-area patients aged between 11 and 17 years were referred within the internal pilot phase (between November 1, 2016, and October 31, 2017). Of these, 150 out of 193 patients (77.7%) were potentially eligible, and 89 out of 150 patients (59.3%) were recruited into the trial: 44 were randomized to FITNET-NHS and 45 to AM treatment (Figure 1). Those recruited came from a wide range of locations across England. A total of 76 out of 89 patients (85.3%) provided their 6-month outcome data, and 75 out of 89 patients (84.2%) provided their 12-month outcome data. A total of 2 out of 89 patients (2.2%) actively chose to withdraw from the trial.

### Adverse Events

No serious adverse events were reported by the 89 participants referred during the pilot phase of the trial. Nonserious health events were reported by 10 of these participants while taking part in the study, and these were reviewed by the principal investigator, sponsor, and an independent data safety monitoring committee. Only 1 adverse event was assessed as *possibly related* to the trial treatment, where a family felt that some CFS/ME symptoms worsened when following treatment recommendations. The family took a break from treatment and then returned to it.

### Exclusions and Declines

Of the 193 referrals, 43 (22.2%) were excluded at eligibility assessment, of whom 20 had a local specialist service; 12 did not have a confirmed CFS/ME diagnosis (including patients referred to be diagnosed by the South West United Kingdom center); 5 had not had the diagnostic blood tests necessary to rule out other causes of fatigue (4 due to needle phobia and 1 was unresponsive to requests by the research nurses to arrange blood tests); 2 were not disabled by their fatigue; 1 was unable to complete web-based modules due to learning difficulties; and 3 had individual exclusion reasons (clinically complicated requiring face-to-face assessment, in treatment with pain services primarily, and already completed trial treatment and rereferred to the service). After these exclusions and declines, 150 potentially eligible referrals remained.

Within the initial period after trial launch, 6 out of 150 (4.0%) potentially eligible patients were incorrectly excluded (by the clinical team) before reaching eligibility assessment by a research nurse. These patients were offered face-to-face clinical treatment as for the normal treatment pathway (outside of the trial). On discovering this, the clinical team was offered extra training, and standard operating procedures for the administrative handling of out-of-area referrals were improved, which ensured no further incorrect exclusions. A total of 5 out of 150 potentially eligible patients (3.3%) were referred by GPs in Wales, where treatment funding arrangements (between the Welsh Health Boards and the CFS/ME center) prevented these patients from entering the trial.

Of the 150 potentially eligible patients, 50 declined to participate. The main reason was that they wanted to be seen face-to-face, with 24 out of 150 (16.0%) potentially eligible patients preferring to travel to the hospital in South West United Kingdom for standard clinical treatment instead of taking part in the trial. Others declined because of symptom improvement (8/150, 5.3% patients), perceived study burden (3/150, 2.0% patients), unwillingness to use Skype (3/150, 2.0% patients), unwilling to wait for the local pediatrician to confirm CFS/ME diagnosis (3/150, 2.0% patients), or other individual reasons/unknown (9/150, 6.0% patients).

### Comparison With Recruitment Projections

In advance of study launch, recruitment projections estimated 286 out-of-area referrals by the end of the first 12 months, expecting 19.9% (57/286) of these to be ineligible and to recruit 67.8% (194/286) of potentially eligible referrals, which would be 156 recruits.

**Figure 2:**
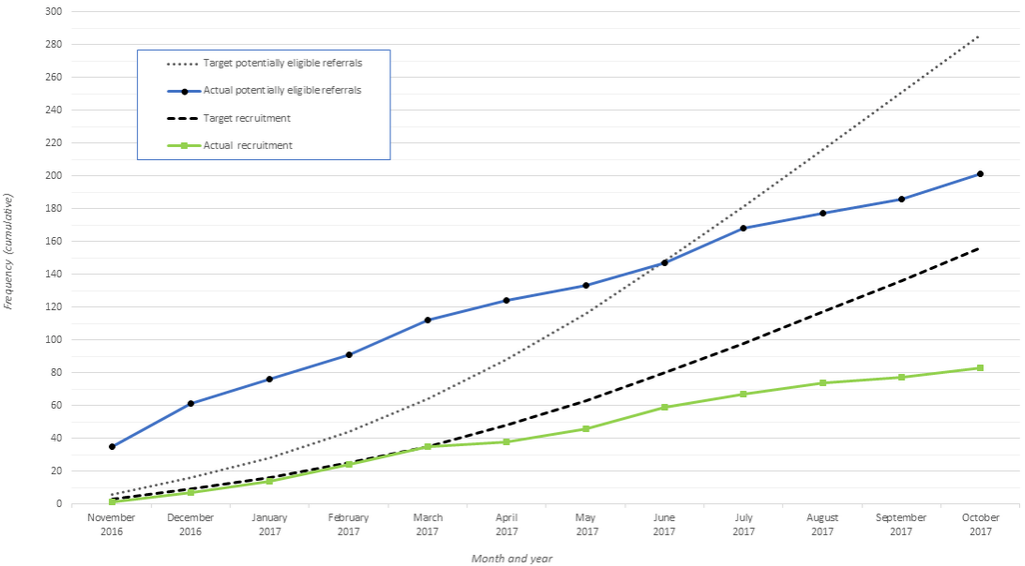
Target versus actual referrals and recruits within the 12-month pilot phase. Note: 6 of the patients who were referred within the pilot phase were recruited later - 5 in November 2017 and 1 in January 2018 (presented in consort, not depicted here as the trial was ongoing).

Projections included an initial lag phase leading up to 35 out-of-area referrals per month. National media coverage at study launch resulted in a surge of referrals (well above projected figures), which waned 6 months later, reducing to under half of the projected out-of-area referrals per month (Figure 2). This had a knock-on effect on recruitment, which suffered a lag of 6 months into the trial (Figure 2).

As described, funding pathway issues prevented patients from Wales from accessing the trial. Similar funding pathway issues existed with other devolved nations (Scotland and Northern Ireland). Due to the distance from the center, these nations were not likely to refer patients for routine (face-to-face) clinical treatment at the service, and because of funding pathway issues, plans to promote the remote treatment opportunity across these regions to gain referrals were not able to proceed, which reduced the pool for potential referrals.

### Qualitative Results

Within the pilot phase, interviews were conducted with 20 families (between February 1, 2017, and October 31, 2017; Table 1): 12 families were in the FITNET-NHS arm and 8 were in the AM arm. This included 18 children (12 females and 6 males, ranging in age from 12 to 17 years) and 22 parents (19 mothers and 3 fathers; 2 interviews included both parents). As all adult interviewees were parents, we refer to them as parents (rather than parents or caregivers) in this section. Children and their parent(s) were given the choice of being interviewed alone or together; 14 were interviewed together; 4 separately; and, for 2 families, only the parent was interviewed. A total of 10 families chose to be interviewed via Skype and 10 over the telephone. In all, 4 families canceled interviews as they were unavailable on the day. Six families contacted declined to be interviewed: 3 were too busy because of school, 1 was too ill at the time, and 2 did not wish to participate. A total of 10 interviews were undertaken with trial staff in person on hospital premises: 2 recruiters, 4 AM therapists, and 4 FITNET-NHS therapists.

**Table 1:** Details of the children and parents interviewed.

ID code	Child age group (years)	Treatment arm (AM^a^: 3 Skype calls; FITNET-NHS^b^: 19 web-based modules)	Interview mode	Parent interviewed	Child/parent interviewed (together/separately)
072	11-12	AM (2nd Skype call)	Skype	Mother	Separately
029	13-15	FITNET-NHS (module 15)	Telephone	Mother	Together
064	13-15	AM (2nd Skype call)	Skype	Mother	Together
082	16-17	FITNET-NHS (module 5)	Telephone	Mother	Together
093	13-15	FITNET-NHS (module 12)	Telephone	Mother	Together
155	13-15	FITNET-NHS (module 15)	Telephone	Mother	Together
198	13-15	FITNET-NHS completed	Skype	Mother and father	Together
209	11-12	AM completed	Skype	Mother	Together
254	13-15	FITNET-NHS (module 17)	Telephone	Mother	Separately
292	16-17	FITNET-NHS (module 15)	Skype	Mother	Together
313	16-17	AM (2nd Skype call)	Telephone	Mother	Parent only
345	13-15	FITNET-NHS (module 15)	Telephone	Mother and father	Together
373	11-12	AM (2nd Skype call)	Skype	Mother	Together
399	13-15	AM (2nd Skype call)	Telephone	Mother	Parent only
401	11-12	FITNET-NHS (module 10)	Skype	Mother	Together
417	11-12	FITNET-NHS (module 15)	Skype	Mother	Together
493	16-17	FITNET-NHS (module 15)	Telephone	Mother	Together
642	16-17	FITNET-NHS (module 14)	Telephone	Father	Together
727	13-15	AM (2nd Skype call)	Skype	Mother	Separately
079	13-15	AM (2nd Skype call)	Skype	Mother	Separately

^a^AM: activity management.

^b^FITNET-NHS: Fatigue in Teenagers on the InterNET in the National Health Service.

### Reasons for Participating in the Trial

Families were positive about taking part in the trial, referring to it as a “lifeline,” and felt they were “lucky” in the context of “absolutely no treatment” elsewhere. They reported problems before the trial: getting a diagnosis, long waiting lists, funding cuts, and only receiving general advice or self-directed treatments with little improvement. Not having to travel to an appointment was seen as beneficial. Families also wanted to “help other future sufferers”.

Well the GP has been absolutely awful, basically saying “Oh nobody knows what chronic fatigue is” and sending him away all the time.

Mother 064

I was willing to try anything.

Child 029

We liked the idea it was online so that we didn't have to travel.

Mother 313

### Provision and Acceptability of Patient Information and the Recruitment Process

The written patient information leaflets were found to be clear, thorough, and acceptable. However, some children were too ill to read them, with parents often explaining the study, and some families felt there was too much information. Shorter leaflets or links to a website with more images were suggested as an additional source of information. The *Frequently Asked Questions* (FAQ)section of the FITNET-NHS website was developed to help this.

…we were drip-fed in the appropriate way; so, we had the written stuff, we could ask questions remotely, and we could ask questions when they called us up. It was done very-very well, the layers of information were appropriate.

Mother 029

I didn’t manage to read through all of it but I did read enough and I understood it and I thought that it would be good to try.

Child 254

Families were particularly happy with telephone recruitment and described the research nurses as “positive,” “understanding,” “empathic,” and “helpful,” allowing them to ask questions. Although some families expressed preferences for treatments, they were often “willing to try anything.” Most participants accepted randomization as part of the research process and understood the need for a “fair” comparison. However, some would have liked to choose their treatment, and a few families did not seem to understand randomization, which was fed back into research nurse training for recruitment calls. Participants preferred web-based consenting and data collection as it was “easy” and there was no need to post paper forms.

Yeah, I think it explained it all and explained it was two different treatments would be available and you’d just be randomly selected for one, pretty straightforward.

Mother 082

I think because [child] has had absolutely no treatment at all or help really from anywhere, she saw this as an opportunity so she was going to take it whichever she was given.

Mother 292

#### The Acceptability (Satisfaction and Adherence) of Interventions

Participants valued individually tailored advice from a *specialist* CFS/ME health professional offered in both treatment arms as they had not had the support before. Families and clinicians commented on both the advantages and disadvantages of web-based treatment.

The good thing is that you do have somebody to be in touch with us more often, because I felt with [child]'s illness that we were sort of left alone, and we see the paediatrician every six months...and there's no treatment that you can have.

Mother 254

#### Acceptability of AM Skype Calls

Skype was found to be easy to use once set up. Some participants felt Skype was as good as being in a face-to-face appointment. They liked that they did not have to travel and felt being in the home environment was beneficial. Therapists also felt that some patients were more comfortable in their home environment. However, some technical difficulties were often encountered with Skype calls. Skype was felt to work less well for shy children, and some families would have preferred to talk to a health professional in person rather than on Skype. Therapists indicated that a face-to-face appointment in a clinic offered a neutral “safe space” for participants to raise issues of importance and that they are more able to pick up on emotions face-to-face. Issues with confidentiality were also discussed, as it was not always clear to therapists “who is in the room” during a Skype call. Therapists also described how some younger children were harder to engage on Skype.

Its [Skype] kinda like face to face...it worked really well and then we didn’t have to travel.

Child 209

Yeah, I mean I think it would better a doctor in person.

Mother 064

Because they are at their home, they are feeling more relaxed. They haven’t had to turn out and travel somewhere, alien environment, being uptight with the traffic. The poor child being exhausted with the travelling, so in many ways you can get a better view of exactly what’s going on for them at home.

Therapist 70005

Sometimes in clinic you’re able to pick up on things that might be a little bit more personal. You see that they’re very upset about something... It’s a little bit more of a safe space to talk about things when they come into clinic, whereas when they’re sitting at home, and it’s just them two and then me, and sometimes that can be a frozen computer screen or just a black screen because Skype hasn’t worked.

Therapist 70008

#### Acceptability of the FITNET-NHS Platform/E-Consultations With a Therapist

Participants liked that they could complete treatment (reading and answering questions on the platform) in their own time rather than having to attend appointments. E-consultations gave them time to think about their answers, and some participants found it easier to talk about personal topics over email. However, others found it difficult to portray things in writing and would have preferred some face-to-face contact. Where therapists received detailed e-consultation replies from patients, they felt they could get “a good picture of them,” whereas some commented that the lack of nonverbal communication made it harder to get to know some patients. Some younger children, and particularly their parents, felt that there was too much reading on the platform, and parents often had to help and clarify the meaning of some text. Age differences were also noted by therapists, with parents more involved as a *coach* with the treatment of younger children. Two therapists also described more engagement from girls and sharing of personal information than boys.

Yeah, I thought it was a bit strange at the beginning but then it was fine. I think sometimes I found it easier to talk to somebody when it wasn’t exactly face to face.

Child 254

Over an email, sometimes it’s quite hard to portray how I feel personally or how I am and how I feel...It’s okay, it’s really convenient, but like I say, face to face you get the whole how I actually am rather than just words how I am.

Child 082

…it’s much more difficult to get to know what a young person might need or how they might be responding when all you’re getting is written information. You’re not having any kind of interaction with the person to know how they’re responding and what their non-verbals might be telling you if you have them in the room. I can see when a young person walks into a clinic room with me, I can see if they’re looking a bit better than they did last week.

Therapist 70006

…the younger ones that you maybe have a bit more emphasis on the parent being a coach alongside you and put a bit more effort in to helping the parents with it and then when they’re older and you know it’s more on the young person themselves.

Therapist 70002

### Changes to the Trial Treatments Based on Qualitative Feedback

#### AM

The qualitative interviews indicated that most participants did not feel 3 Skype calls were enough. Clinicians did not feel that the 2 treatment arms were equal and failed to match the current standard for usual care, which had increased to 6 calls per patient since the trial was designed. In response to this feedback, the AM arm was changed to allow up to 6 sessions (submitted as a substantial amendment, gaining ethical approval in October 2017; Multimedia Appendix 1). Other small changes to treatment were made based on feedback, such as splitting the first Skype (assessment) call into 2 shorter sessions and sending a summary email of the Skype call to families to summarize agreed actions.

I personally think there should be more [AM sessions] because obviously you are just getting going.

Mother 373

I do worry a little bit about how equal the two arms are. It does feel like people do FITNET for good or ill really, have a lot more to do...the activity management arm is three Skype sessions...But it feels like they don’t feel comparable in terms of therapist input, which can be a factor in itself in terms of outcomes I would imagine.

Therapist 70004

#### FITNET-NHS

Several smaller changes were made to the platform based on feedback received during the pilot phase of the trial (Multimedia Appendix 2). For example, a time-out function existed within the platform for data protection, although this meant that the platform often timed out while a participant was writing a lengthy message (because of remaining on 1 page). Changes were made to warn participants of this. Changes to wording on the platform to clarify meaning were made and clearer instructions accompanied the diaries.

[Session] timed out so I had to write the whole email again.

Child 642

You almost need instructions to understand it [diaries].

Child 401

## Discussion

### Principal Findings

This is the first pilot of specialist web-based treatments for CFS/ME in young people in the United Kingdom, representing the first 12 months of an ongoing large, full national RCT. We demonstrated that it is feasible to recruit children with CFS/ME (and their parents or caregivers) remotely (via telephone screening and web-based consent) and retain them in a trial providing web-based specialist treatment for pediatric CFS/ME.

### Strengths and Limitations

A major strength of this study is its use of entirely web-based treatment to enable families across the United Kingdom to gain treatment at home delivered from 1 specialist service. Many families appreciated the opportunity to gain access to treatment, especially when they otherwise would have no access to specialist care. In contrast to the Dutch study, the FITNET-NHS trial used an active specialist treatment comparison group (AM via Skype), rather than treatment as usual or waiting list control, as specifically recommended for this trial by the funders. This is likely to have implications for the results of the full-scale trial (in terms of relative treatment effectiveness) and may also have contributed to the families’ willingness to enter the trial, as all recruited participants were offered specialist treatment. The recruitment rates were good, and the families were positive about the remote recruitment processes.

Another strength is the integration of qualitative methods into this RCT to improve recruitment and optimize the interventions in the pilot phase [12,15,16]. These methods helped us understand issues relevant to children and young people and make changes for the full study, including increasing AM sessions in line with usual care and facilitating training of research nurses to improve the recruitment processes. A range of young people of different ages were interviewed from both arms; however, fewer males and fathers were interviewed, which decreased the ability to explore any differences in acceptability [10]. Although we did not interview those declining the trial (which may have been useful to illuminate the reasons), we kept records of the main reasons for declining.

A limitation is the lower recruitment compared with projections. We planned to recruit 156 participants in the in-pilot phase, representing 68% of the potentially eligible patients (expected to be 229) identified from out-of-area referrals aged between 11 and 17 years (expected n=286). We recruited 89 out of 150 participants, representing 59% of the potentially eligible young people identified from 193 referrals. The potentially eligible denominator included 11 referred patients who were excluded due to funding issues or in error (omitting these from the denominator gives us 89 participants recruited out of 139, representing 64% uptake, which is closer to the original projections). The main issue with achieving recruitment targets was the lower than expected number of out-of-area referrals received. Although the CFS/ME service has always accepted out-of-area referrals, most were local referrals. Recruitment projections were based on reaching national pediatric CFS/ME populations at high volumes, made possible by our innovative methodology using remote processes for recruitment and treatment delivery. Increasing out-of-area referrals was dependent upon maintaining clinician awareness of the trial across the United Kingdom, which proved to be more challenging than expected. The loss of the devolved nations as potential referral sites was part of this picture. In response, detailed plans to increase awareness of the trial across England were put into place to boost referral rates (including drawing on the National Institute for Health Research [NIHR] Clinical Research Network support to publicize the research across the United Kingdom, developing a clinician-facing video to disseminate, and presenting at national GP and pediatrician meetings). These plans, together with evidence of the acceptability of treatments and good retention were reviewed by the independent oversight groups (Trial Steering Committee [on October 3, 2017] and Data Safety Monitoring Committee [on September 5, 2017]) and the funding body (NIHR Health Technology Assessment [HTA]). These groups recommended that the study proceed to full trial after the end of the pilot phase (from November 1, 2017).

### Results in the Context of Previous Literature

Recruitment rates of families referred to the service were good, with over half of potentially eligible referrals enrolled in the trial. Of the families declining to take part, approximately half opted to travel for face-to-face treatment rather than accepting web-based treatment. This is in line with other research in adults, which had similar rates of uptake [17]. By comparison, not having to travel for treatment was perceived as a benefit by those who took part. Retention at 6 months was good (76/89, 85%) and was maintained up to 12 months, with only 2 participants requesting withdrawal from the trial.

The 2 web-based treatment options—FITNET-NHS (web-based CBT) and Skype-delivered AM—are acceptable to young people with CFS/ME and their parents/caregivers. Access to tailored advice from a *specialist* CFS/ME health professional was particularly valuable to participants, and therapist support has been found to be important for increasing engagement and adherence to digital interventions in mental health [18]. Both advantages (eg, less travel and home environment) and disadvantages (eg, technical problems with Skype and preference for face-to-face contact) were discussed by participants. Other research identified a similar mix of positive and negative patient experiences of CFS/ME treatment delivery via videoconferencing [19].

This study builds on the promising results of the Dutch trial of the FITNET program [5] by demonstrating the potential for the UK-adaptation of the web-based CBT intervention to be used within the NHS. The full RCT is currently ongoing, with recruitment due to be completed by October 31, 2020 (with follow-up data collection to continue for 12 months beyond this). The results of the full trial will indicate whether the FITNET-NHS treatment is effective and cost-effective when compared with web-based AM, which will inform treatment recommendations going forward.

### Conclusions

It is possible to recruit and retain families in a CFS/ME treatment trial using telephone and web-based methods. Many families are willing to accept web-based treatment. This supports the potential for remote delivery of treatment and trials for different clinical populations who cannot access local services where such an approach could benefit patients.

## Appendix

Multimedia Appendix 1 Details of approved study amendments.

Multimedia Appendix 2 Changes made to the FITNET-NHS Platform.

Multimedia Appendix 3 EHEALTH CONSORT checklist (v1.6.1).

## Abbreviations

AM: activity management
CBT: cognitive behavioral therapy
CFS/ME: chronic fatigue syndrome/myalgic encephalomyelitis
FITNET: Fatigue in Teenagers on the InterNET
FITNET-NHS: Fatigue in Teenagers on the InterNET in the National Health Service
GP: general practitioner
HTA: Health Technology Assessment
NHS: National Health Service
NIHR: National Institute for Health Research
RCT: randomized controlled trial
SF-36-PFS: Short Form Health Survey Physical Function Subscale
TMG: Trial Management Group
