# Supplementary material for: Recruiting Adolescents With Chronic Fatigue Syndrome/Myalgic Encephalomyelitis to Internet-Delivered Therapy: Internal Pilot Within a Randomized Controlled Trial
Source: J Med Internet Res. 2020 Aug 12;22(8):e17768. doi: 10.2196/17768 (PMC7450376; doi:10.2196/17768)
Supplement: Multimedia Appendix 1 [file jmir_v22i8e17768_app1.docx]

## Appendix 1: Details of approved study amendments

*FITNET-NHS REC reference: 16/SW/0268; Protocol number: 2571; IRAS project ID: 211202*

| 1. **Substantial amendment 1**   *Approved by South West – Frenchay, Research Ethics Committee: 19/10/2017*  *Approved by Health Research Authority: 23/10/2017* |
| --- |
| Summary of changes:  Substantial:   - Activity Management increased to six (rather than 3) sessions   *Reasons:*  *- The average number of sessions offered by the Bath clinical team as standard (outside of the trial) is 4.8 follow-ups after a first appt (i.e. 5.8 total). The theoretical model is to see if FITNET-NHS is effective and cost-effective compared to a form of treatment as usual (TAU). 3 AM sessions is less than TAU.*  *- The FITNET Trial protocol does not stop AM participants from re-referring to the service after 3 sessions for face to face treatment (outside of the trial).*  *- Feedback is that three is seen as not enough (from recruitment calls and treatment response). i.e. is unacceptable for families that AM is so brief, and we feel it is unethical to continue at 3 sessions for this reason*  *- The TMG, TSC, Sponsor and funder (HTA) have all been consulted and approve of this plan.*   - Allow researchers to analyse content of therapeutic emails sent/received within the FITNET-NHS platform.   *Examining the content of the emails exchanged between the patients (participants) and the therapists will enable us to explore a variety of process issues in internet-delivered CBT with adolescents with CFS/ME. Potentially, we could explore therapist behaviours, patient behaviours, parent behaviours (in the emails they exchange with the therapists) and inter-relational patterns between the therapists and the patients/parents. This is important as it could help us to identify how to most effectively utilise an online therapy approach with this patient group. For example, it could help us to identify early warning signs of disengagement from therapy and what therapist strategies are most effective in terms of addressing this.*  Details of changes to key documents:  Protocol v5: updated to show 6 activity management sessions in the following sections: Section 1. Summary; Section 4.2, Figure 2; Section 4.6.1 (activity management (comparator))  Information sheets (PILs) for participants updated to show 6 Activity Management sessions and that the content of therapeutic emails will be analysed. Updated documents: PIL v4.0 for 11-15yrs; PIL v4.0 for 16-17yrs; PIL v4.0 for parent/carer  Consent forms updated to include optional consent for analysing content of therapeutic emails. Updated documents: Consent to study for 11-15 yrs v4; Consent to study for 15-16 yrs v4  Additional minor amendments submitted with substantial amendment 1:  *(sponsor approved these and confirmed that there is no need for REC)*  1. The following details in the protocol were amended:   - - - 1. Simon Price and Mary-Jane Willows have now left the trial team. Sarah Baos has been replaced by Emma Anderson as Trial Manager. Protocol section amended: ‘Research Team Contact Details’ (p1-2)       2. Clarified duration of initial Activity Management assessment. Protocol section amended: 4.6.1 'Activity Management (comparator)'       3. Data collection – added the option of contacting participants by text and/or letter. Protocol section amended: 4.11.1 'Data collection using REDCap'       4. The Activity Management treatment can be delivered by specialist clinicians from the Bath CFS/ME team that are from different professional backgrounds, not just Occupational Therapists.Protocol sections amended: Section 1: Summary; Section 4.6.1. Activity Management (Comparator); Every further instance where "Occupational Therapist" is stated as the Activity Management therapist has been changed to "clinician" or "specialist clinician" for clarity.   2. The health economics questionnaires are filled in by one parent, and asks for answers to questions about a second parent involved in the child’s care. We updated the questionnaire to include a question as to whether they feel able to answer questions on the second parent’s behalf. A ‘No’ response hides those questions. NB: This is for the convenience of the participating parent, especially to make it easier for families in which the parents are separated. Updated documents:  Health Economics_baseline questionnaire_parental_v2  Health Economics_fu 3m questionnaire_parental_v2  Health Economics_fu 6m questionnaire_parental_v2  Health Economics_fu 12m questionnaire_parental_v2  3. Following discussion by the TMG, it was agreed that the current ethnicity categories captured at baseline were unhelpful for analysis (in particular having British first and then separate categories for English, Irish etc followed by "any other white background"). These have been simplified in line with ONS recommended categories. Updated document: Baseline questionnaires_v2 (tracked changes)  4. The information leaflets for participants and parents have been amended for clarity about the description of trial treatments and the AM assessment appointment. This is in response to participant feedback.Updated documents:  FITNET-NHS PIL_11-15 yrs_v4 (tracked changes)  FITNET-NHS PIL_16-17 yrs_v4 (tracked changes)  FITNET-NHS PIL_parent carer_v4 (tracked changes)   1. Consent form updated to detail the new version number (and date) of information sheets - no need for ethical review of consent form as no other change - clarification only. (Updated version number consent forms supplied).   Two points of clarification:   - The protocol states that adherence to treatment is defined as 80% completion, without stating how this will be measured. On discussion with the clinical team, health economist and Trial Management Group, we have agreed that therapists will make this rating as a clinical judgement of treatment completion using three categories (no treatment, partial treatment and treatment completion i.e. 80% or above), based on assessing how much the participant worked through what was expected of the modules (FITNET-NHS arm), or activity management sessions offered. - We changed the wording of one of the routine screening questions used by the clinical team in their patient assessment for clarity. NB: This was a clinical site-wide change to routine assessment practices, and is not research-specific, therefore it is not a study amendment.   - The previous wording is: Q2.7 No clinical evidence of other causes of fatigue True of False  - The new wording is: Q2.7 No clinical evidence of other principal/primary/main causes of fatigue True or False |
