# Supplementary material for: Recruiting Adolescents With Chronic Fatigue Syndrome/Myalgic Encephalomyelitis to Internet-Delivered Therapy: Internal Pilot Within a Randomized Controlled Trial
Source: J Med Internet Res. 2020 Aug 12;22(8):e17768. doi: 10.2196/17768 (PMC7450376; doi:10.2196/17768)
Supplement: Multimedia Appendix 2 [file jmir_v22i8e17768_app2.docx]

## Appendix 2: Changes made to the FITNET-NHS Platform

| **Origin** | **Date** | **Findings** | **Changed/ Wish** | **Changes Made** | **Date Changed** |
| --- | --- | --- | --- | --- | --- |
| **Participant Interviews** | **April 2017** | Families reported some questions and text on the platform is not clear (couldn’t remember specific sections in interviews). | **Changed** | FITNET therapist will include a section in initial email to prompt patients to report any problems with content as they come across them. | Several changes made for specific text requests at different times |
| **Participant Interviews** | **April 2017** | Several patients reported not understanding how to fill out diaries or what they should be seeing. | **Changed**  (after pilot phase) | Need guidance sheets / videos of what to expect/ see/ do on platform | 03/10/2018 |
| **Clinician Interviews** | **April 2017** | FITNET therapists reported patients misinterpret- Section 1.5: 1*. Can you describe yesterday to me? From the moment you woke up and got out of bed to the moment you went to bed at night.*  Some external people have just answered this very literally with a No or Yes! | **Changed** | Text on platform changed to  *“1. Tell me about yesterday, from the moment you woke up and got out of bed to the moment you went to bed at night.”* | 22/9/17 |
| **Clinician Interviews** | **April 2017** | FITNET patient wanted to withdraw due to wording on platform (section 2.6- 2.19) they felt indicated they do not have a physical illness. | **Not changed** | C.I decided not to change | n/a |
| **Clinician Interviews** | **April 2017** | Therapists need to be able to access all message history for each other’s patients in case of illness or patient needing to be transferred to another therapist | **Changed** | Allowed all messages to be seen by all therapists | 16/6/2017 |
| **Participant Interviews** | **April 2017** | Need to make certain questions clearer:  e.g.  Section 1.7 question 5= does it mean in person or on social media?  Section 13.3 questions 1 and 2- [child] felt that question 2 was the same answer as question 1. | **Changed** | Section 1.7 question clarified on platform:  *“5. When was the last time you spoke to your best friend and was this in person?”*  Section 13.3 question 1 & 2 clarified | 22/9/17 |
| **Participant Interviews** | **April 2017** | Patients found the use of the word 'camps' weird. | **Changed** | Word ‘camps’ on platform changed to ‘school trips’. | 22/9/17 |
| **Participant Interviews** | **April 2017** | Examples in chapter e.g. bar charts (section 8.5 &10.6) do not correspond with how the activity diary needs to be completed (one horizontal, one vertical). Patients found this confusing. | **Changed** | Added examples to platform that match the diaries. | 22/9/17 |
| **Participant Interviews** | **April 2017** | Sleep diary needs to be over 3 days | **Changed** | Added ability to view sleep diary entry history. | 3/7/17 (therapist view);  1/8/17 (family view) |
| **Participant Interviews** | **April 2017** | Parents would like reminders sent to an external email address to the platform (e.g hotmail) to remind them to log in. | **Changed** (after pilot phase) | Added ability for therapists to send an automated message from the platform to an external email address to remind parents to check the platform | 18/3/2019 |
| **Clinician Interviews** | **May 2017** | Messages on platform need to be chunked up per patient rather than appearing in a long list with emails from other therapists/ patients | **Changed** | Added filter/ search function to messages list and ability for all therapists to read all messages | 16/6/17 |
| **Clinician Interviews**  **Participant Interviews** | **May 2017**  **July 2017** | Therapists would like to be able to attach diagrams to email | **Not changed** | Not addressed due to level of complication (and risk) this change would bring to the platform | n/a |
| **Clinician Interviews** | **May 2017** | Therapists would like to be able to format messages (bold, underline etc…) | **Changed**  (shortly after pilot phase) | Changed | 21/12/2017 |
| **Clinician Interviews** | **May 2017** | There is a problem with messages timing out.  The platform was designed with a 30 minute time-out (auto-logout after 30 minutes’ inactivity) feature for data protection. However, if a participant stayed on one page for longer than 30 minutes, it would not register this as activity and the time-out would kick in, which affected participants spending a long time writing a message to their therapist as the time-out wiped the information. | **Changed** | Text added on platform (Chapter 1) to warn patients of time-out.  Pop-up message function was later created to warn participants to save their messages 5 minutes in advance of the time-out.  Later again, this issue was resolved by changing the IT codes to recognise key strokes (while staying on one page) as activity that prevents the time-out. | 19/7/17  19/1/2018  11/3/2019 |
| **Clinician Interviews** | **May 2017** | Activity diary needs to display longer history of activity | **Changed**  (after pilot phase) | Changed | 04/6/2018 |
| **Clinician Interviews** | **May 2017** | Word limit on emails- therapists having to split emails into two. | **Changed** | Word limit removed to allow longer messages | 19/7/17 |
| **Clinician Interviews** | **May 2017** | Can’t record 0 hours in the school diary | **Changed** | Error fixed | 31/7/17 |
| **Clinician Interviews** | **May 2017** | Don’t need response to fatigue diary- already have a ‘helpful thoughts’ diary. | **Changed** (after pilot phase) | Fatigue diary deleted | 18/7/2018 |
| **Clinician Interviews** | **May 2017** | Chat function not useful | **Changed** | Chat function removed | 26/10/17 |
| **Clinician Interviews** | **May 2017** | Section on making a school/ social plan needs to be more specific. It requires a patient to plan ‘on Monday I will’ but patients have been responding “I will do the best I can” | **Changed** | Added the following text after the school goals question (How can you reach these goals?):  “(i.e. what are your plans for how to reach them)?” | 22/9/17 |
| **Clinician Interviews** | **May 2017** | Therapist want to be able to see if patient is logging in | **Changed** | Therapist able to see date last logged in | 01/08/17 |
| **Clinician Interviews** | **May 2017** | Text on chapter 15 inconsistent- one page says you are ‘walking twice a day’ and then the next page says, you are ‘walking once a day’. | **Changed** | Text edit made for clarity | 13/6/2017 |
